# Prognostic Value of Neutrophil-to-Lymphocyte Ratio for Adverse Outcomes in Pediatric Congenital Heart Disease Surgery

**DOI:** 10.1055/s-0046-1827862

**Published:** 2026-09-09

**Authors:** Amna Hassan Mohamed, Mohamed Salah Khalil Salih, Abubakar ElKabashi, Abdelmoiz El. A. Mohamed, Fatima Salah Khalil Salih, Sara Ali Ishag Ahmed, Hussein Ahmed, Mohammed Haroun, Eman Eissa, Sitelgeel A.M. Alawad, Yahia Eltayeb, Sagad O. O. Mohamed

**Affiliations:** 1 Faculty of Medicine, University of Khartoum, Khartoum, Sudan; 2 Internal Medicine Department, NYC Health + Hospitals/Harlem Hospital Center, Columbia University Vagelos College of Physicians and Surgeons, New York, United States; 3 General Surgery Department, Omdurman Islamic University, Omdurman, Sudan; 4 General Surgery Department, University of Science and Technology, Khartoum, Sudan; 5 Internal Medicine Department, Ascension Saint Joseph Hospital, Chicago, Illinois, United States; 6 Paediatrics Department, Maidstone and Tunbridge Wells NHS Trust, Maidstone, United Kingdom; 7 Paediatric Cardiology Department, Alder Hey Children's Hospital, Liverpool, United Kingdom

**Keywords:** neutrophil-to-lymphocyte ratio, congenital heart disease, pediatric cardiac surgery, inflammatory biomarkers, prognosis, postoperative complications, mortality, risk stratification

## Abstract

Perioperative inflammatory response is implicated in postoperative complications in pediatric patients undergoing congenital heart disease (CHD) surgery. Neutrophil-to-lymphocyte ratio (NLR) is an inflammatory biomarker that may indicate systemic stress and predict postoperative complications. This systematic review synthesizes the existing evidence on perioperative NLR in pediatric CHD surgery and its relationship with mortality and postoperative complications. A systematic review was conducted according to PRISMA guidelines. The search was performed in PubMed, Web of Science, Embase, and ProQuest. Pooled standardized mean differences (SMDs) and correlation coefficients ( *r* ) with 95% confidence intervals (CI) were calculated to investigate the differences in NLR values and their relationships with postoperative outcomes in pediatric CHD surgery. A total of 16 studies were selected for the systematic review. Of them, all 16 were included in the meta-analyses. Perioperative NLR showed a trend toward higher values in patients with adverse outcomes; however, none of the pooled estimates reached statistical significance. For preoperative NLR, the pooled effect size (SMD) was 0.63 (95% CI: −0.27–1.52, *p* = 0.17), and for postoperative NLR, it was 0.74 (95% CI: −0.27–1.75, *p* = 0.15). Meta-analyses of correlations revealed that NLR was not significantly correlated with intensive care unit length of stay ( *r* = 0.39, *p* = 0.19), hospital length of stay ( *r* = 0.43, *p* = 0.15), duration of mechanical ventilation ( *r* = 0.43, *p* = 0.28), or overall postoperative complications (SMD = 0.55, *p* = 0.094). Perioperative NLR in pediatric CHD surgery shows a trend toward higher values in patients with adverse outcomes but does not demonstrate a statistically significant association with mortality or complications. NLR reflects the inflammatory response but may not be sufficient as an independent predictor in pediatric patients undergoing cardiac surgery. Its clinical utility may be greater when used as part of combined risk assessment strategies.

## Introduction

Congenital heart disease (CHD) remains the most common congenital anomaly worldwide, affecting approximately 8 to 10 per 1,000 live births and representing a major cause of morbidity and mortality in children. 1 Advances in surgical techniques, cardiopulmonary bypass (CPB) technology, and perioperative intensive care have markedly improved survival in pediatric cardiac surgery over the past decades. Nevertheless, postoperative complications such as low cardiac output syndrome, prolonged mechanical ventilation, infection, and mortality remain significant concerns, particularly in complex congenital lesions. 2 Identifying reliable biomarkers that can predict adverse outcomes in this population may assist clinicians in risk stratification and perioperative management.

Inflammation plays a central role in the pathophysiology of perioperative complications following cardiac surgery. The use of CPB, surgical trauma, ischemia-reperfusion injury, and exposure to artificial surfaces can trigger a systemic inflammatory response that contributes to postoperative organ dysfunction. 3 4 In recent years, several inflammatory biomarkers have been investigated for their prognostic value in cardiovascular disease and cardiac surgery.

Among these markers, the neutrophil-to-lymphocyte ratio (NLR) has gained attention as a simple and inexpensive indicator of systemic inflammation derived from routine blood tests. NLR reflects the balance between neutrophil-mediated innate inflammatory responses and lymphocyte-mediated regulatory or adaptive immune pathways. 3 Elevated NLR levels have been associated with adverse outcomes in a wide range of cardiovascular conditions, including coronary artery disease, heart failure, and adult cardiac surgery. 5 6 Several studies have demonstrated that increased perioperative NLR is linked with higher mortality, longer intensive care unit (ICU) stay, and increased postoperative complications in adult patients undergoing cardiac procedures. 5 6

Despite growing evidence in adult populations, the prognostic significance of NLR in pediatric cardiac surgery remains less clearly defined. Children with CHD differ from adults in terms of underlying pathophysiology, immune response, and surgical complexity, which may influence inflammatory biomarkers and their predictive value. 7

Given these uncertainties, a comprehensive evaluation of the available evidence is warranted. This review aims to assess the prognostic value of perioperative NLR in predicting mortality and adverse outcomes among pediatric patients undergoing surgery for CHD.

## Methods

### Search Approach and Study Inclusion Criteria

This systematic review was conducted in accordance with PRISMA guidelines. 8 The review was prospectively registered on PROSPERO (CRD420261322058). The Population, Exposure, Comparison, Outcome (PECO) model was used to structure the review question. The population consisted of pediatric patients undergoing cardiac surgery (<18 years). The exposure was elevated perioperative (preoperative or postoperative) NLR values. The comparator included patients with lower NLR values. The outcome of interest was the development of any adverse postoperative outcome.

A systematic literature search was carried out across PubMed, Web of Science, Embase, and ProQuest, with no restrictions on publication date or geographic origin. The database search covered literature from inception through February 2026, using a combination of terms related to NLR and CHD surgery ( Supplementary Material S1 , available in the online version only). Reference lists of included articles were hand-searched to capture any missed studies. All retrieved records were imported into Rayyan ( https://www.rayyan.ai ) for deduplication and preliminary title/abstract screening. 9

Study selection proceeded in two stages: initial screening of titles and abstracts followed by full-text review, both performed independently by three reviewers. Eligible study designs included cross-sectional, case-control, and cohort studies reporting NLR data. Case reports, reviews, editorials, conference abstracts, and studies with insufficient outcome data were excluded.

### Quality Assessment and Data Extraction

Risk of bias was appraised using the Quality in Prognosis Studies (QUIPS) tool. 10 The QUIPS tool evaluates six domains: study participation, study attrition, prognostic factor measurement, outcome measurement, study confounding, and statistical analysis and reporting. Each domain was rated as low, moderate, or high risk of bias for all included studies. An overall risk-of-bias judgment for each study was determined by considering the ratings across the six domains.

Extracted variables included author, publication year, country, sample size, patient characteristics, conditions and type of cardiac surgery, perioperative NLR values, and effect estimates related to postoperative outcomes. Where studies reported medians and interquartile ranges, means and standard deviations were estimated using the Wan et al conversion method. 11 Discrepancies between reviewers were resolved by consensus.

### Statistical Analysis

All primary analyses were performed in Jamovi ( https://www.jamovi.org ). Pooled standardized mean differences (SMDs) and correlation coefficients, with 95% confidence intervals (CIs), were calculated to evaluate associations between NLR and adverse postoperative outcomes. The random-effects meta-analysis model (restricted maximum-likelihood estimator) was employed throughout to accommodate between-study heterogeneity, which was quantified using the *I* ^2^ statistic. Publication bias was assessed using Begg's and Egger's tests, supplemented by visual inspection of funnel plots. A significance threshold of 0.05 was applied to all analyses.

## Results

### Studies Characteristics

**Fig. 1:**
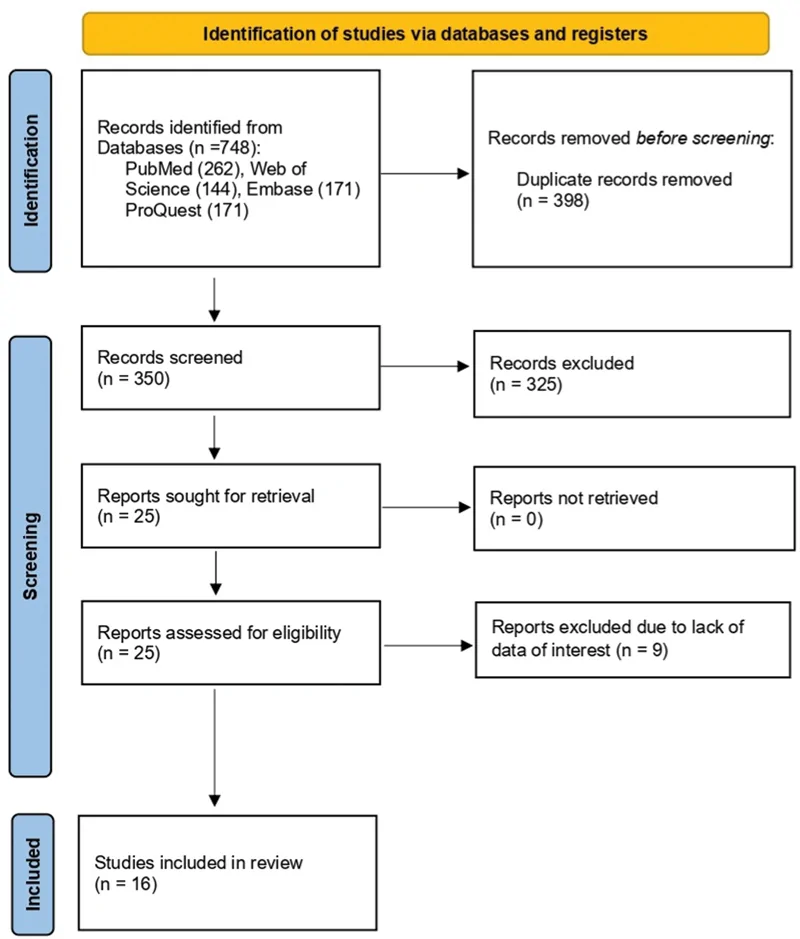
Flow chart of the study selection process.

A total of 748 records were identified through database searching. After removal of 398 duplicate records, 350 unique studies remained and were screened based on titles and abstracts. Of these, 325 records were excluded due to irrelevance and 9 reports were excluded due to lack of data of interest. Lastly, a total of 16 studies, comprising 3,732 pediatric participants, were retrieved and included in the final systematic review 12 13 14 15 16 17 18 19 20 21 22 23 24 25 26 27 ( Fig. 1 ).

**Fig. 2:**
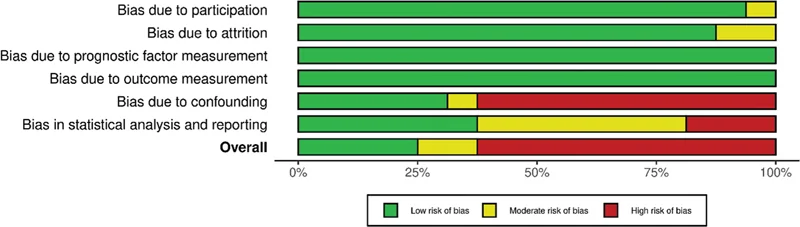
Risk of bias assessment.

The studies were conducted across diverse geographic regions, including Asia, North America, and the Middle East. Patient ages varied widely, with most cohorts consisting of infants. The studies included patients undergoing surgery for CHD, with common conditions including septal defects, tetralogy of Fallot, and complex anomalies ( Table 1 ). Risk of bias of the included studies was evaluated using the QUIPS tool. Most studies demonstrated a low risk of bias in the domains of study participation, study attrition, prognostic factor measurement, and outcome measurement. In contrast, the domains of confounding and statistical analysis showed variability, with several studies rated as having moderate or high risk of bias ( Fig. 2 ).

**Table 1:** Summary of the included studies

Study	Country	Total *N*	Age	Male %	Conditions and type of surgery
Arslanoğlu et al (2021) 12	Turkey	67	30.59 ± 147.26 mo	55.7%	Pediatric CHD surgery requiring post-op ECMO
Entezari Farahabadi et al (2025) 13	Iran	457	25.84 ± 36.9 mo	51%	Pediatric CHD surgery requiring open-heart surgery with CPB
Iliopoulos et al (2020) 14	United States	47	4.1 (0.2–7.6) mo	59.6%	Conotruncal anomalies, left-sided obstruction, valve lesions, and other CHD requiring surgery with CPB
İştar et al (2025) 15	Turkey	70	(1–48) mo	48.6%	Pediatric CHD surgery requiring cardiac surgery with CPB
Kern-Allely et al (2024) 16	Iran	416	27.2 months (2 d–17 y)	NA	Pediatric CHD surgery requiring cardiac surgery with CPB
Kılıç and Balik (2023) 17	Turkey	14	Mean GA 25.7 ± 2.0 wk	42.9%	Patent ductus arteriosus (PDA) requiring PDA ligation (off-pump)
Kumar et al (2024) 18	India	219	42.2 ± 28.6 mo	58.9%	Pediatric CHD surgery requiring cardiac surgery with CPB
Laila et al (2024) 19	Indonesia	90	28 mo (1 mo–18 y)	56.7%	Pediatric CHD surgery requiring cardiac surgery with CPB
Raharjo et al (2024) 20	Indonesia	156	7.13 ± 4.89 y	40.4%	ASD, VSD, sub-aortic stenosis, and Ebstein anomaly requiring open-heart surgery
Savluk et al (2019) 21	Japan	53	(16.5 ± 1.4) and (16.1 ± 1.8) d	71.7%	Hypoplastic left heart syndrome requiring Norwood stage 1 procedure
Sharifi et al (2023) 22	Iran	275	32.54 ± 37.4 mo	NA	Pediatric CHD surgery requiring open-heart surgery
Siagian et al (2025) 23	Indonesia	387	44.8 (21.8–99.8) mo	56.6%	Tetralogy of Fallot repair
Şişli et al (2016) 24	Turkey	51	2 y (median)	58.8%	Tetralogy of Fallot repair
Walian et al (2022) 25	India	1,040	24.91 mo	60.29%	Acyanotic CHD (ASD, VSD, AVSD) with PAH requiring cardiac surgery with CPB
Yang et al (2022) 26	China	200	2.4 y	56%	CHD with and without post-op PAH requiring cardiac surgery
Yin et al (2021) 27	China	190	18.38 mo (median)	54.7%	Simple and complex CHD with PAH requiring open-heart surgery with CPB

Abbreviations: ASD, atrial septal defect; AVSD, atrioventricular septal defect; CHD, congenital heart disease; CPB, cardiopulmonary bypass; ECMO, extracorporeal membrane oxygenation; GA, gestational age; PAH, pulmonary arterial hypertension; VSD, ventricular septal defect.

Across the included studies, perioperative NLR demonstrated inconsistent and inconclusive associations with clinical outcomes in pediatric cardiac surgery. While several studies reported no significant relationship between NLR and mortality or postoperative recovery parameters, others identified elevated perioperative NLR as predictors of adverse outcomes, including prolonged mechanical ventilation, extended ICU stay, low cardiac output syndrome, and mortality. Postoperative and serial NLR measurements appeared to have stronger predictive value in some studies. However, a number of studies showed only weak or nonsignificant correlations ( Table 2 ).

**Table 2:** Summary of the results and outcomes related to NLR

Study	Outcomes related to NLR
Arslanoğlu et al (2021) 12	No significant association between NLR and mortality or ECMO duration.
Entezari Farahabadi et al (2025) 13	Post-op NLR associated with pulmonary complications post-cardiac surgery.
Iliopoulos et al (2020) 14	Higher pre-op NLR associated with higher odds of low cardiac output syndrome; no significant association with inotrope-free state time.
İştar et al (2025) 15	Pre-op NLR cut-off of 1.14 (AUC = 0.75) associated with prolonged ventilation (>72 hours), longer ICU stay, and hospital stay. NLR values on POD3 (AUC = 0.74) and POD6 (AUC = 0.78) also demonstrated strong predictive ability for these outcomes.
Kern-Allely et al (2024) 16	Post-op NLR weakly correlated with ventilation duration, but not with mortality or ICU stay. NLR Change Index (NCI) showed significant association with ICU and ventilation.
Kılıç and Balik (2023) 17	Post-op NLR was not correlated with mortality, postoperative length of stay, and postoperative mechanical ventilation
Kumar et al (2024) 18	Higher pre-op NLR independently predicted poor outcomes (prolonged ventilation, ICU stay, low cardiac output syndrome, and AKI).
Laila et al (2024) 19	Pre-op and post-op NLR (0, 4, and 8 hours) significantly associated with low cardiac output syndrome.
Raharjo et al (2024) 20	Significant difference in the NLR between patients who experienced mortality and those who did not. The increase in NLR had a significant relationship with CPB time, extubation, and ICU length of stay
Savluk et al (2019) 21	High NLR was found to be associated with an increased risk of death. NLR predicted mortality with a sensitivity of 78% and a specificity of 65%.
Sharifi et al (2023) 22	The NLR was higher in those who survived, while the deceased patients had an extended duration of hospitalization and longer CPB time
Siagian et al (2025) 23	Higher preoperative NLR was observed in patients with mortality and complications, though the association lacked statistical significance.
Şişli et al (2016) 24	Pre-op NLR did not significantly predict post-op morbidity.
Walian et al (2022) 25	Pre-op NLR was an independent predictor of poor outcomes (mortality, sepsis, renal failure).
Yang et al (2022) 26	NLR was significantly higher in the post-op pulmonary arterial hypertension group.
Yin et al (2021) 27	Pre-op and post-op NLR weakly correlated with stay time and prolonged mechanical ventilation (>24, 48, 72 hours).

Abbreviations: AKI, acute kidney injury; AUC, area under the curve; CPB, cardiopulmonary bypass; ECMO, extracorporeal membrane oxygenation; ICU, intensive care unit; NLR, neutrophil-to-lymphocyte ratio.

### Association Between NLR and Mortality

**Fig. 3:**
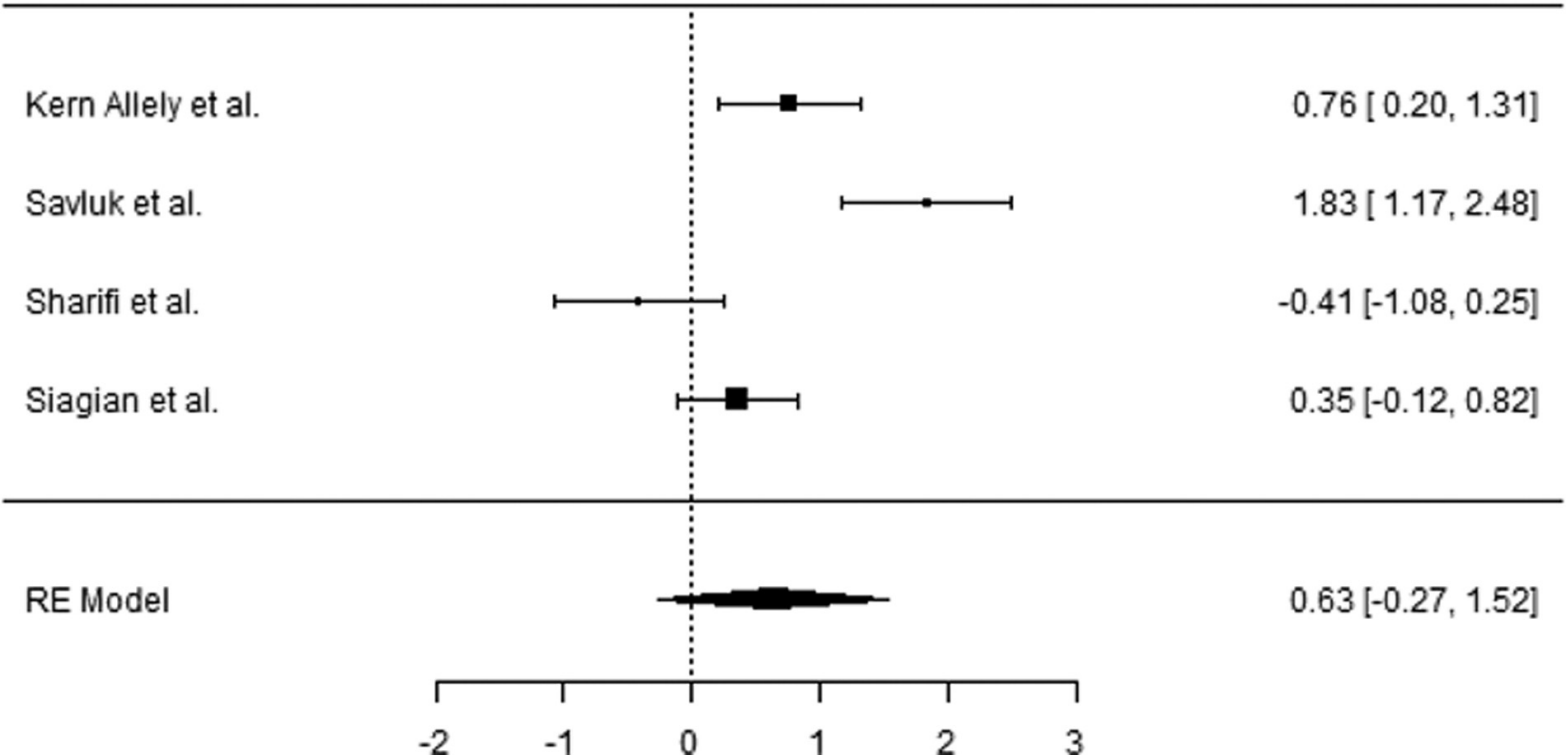
Forest plot of preoperative NLR and mortality. NLR, neutrophil-to-lymphocyte ratio.

Across all analyses, perioperative NLR (both preoperative and postoperative) showed a trend toward higher values in patients with mortality; however, none of the pooled estimates reached statistical significance. Regarding preoperative NLR, a total of four studies were included in the analysis assessing NLR and mortality. The meta-analysis revealed a pooled SMD of 0.63 (95% CI: −0.27 to 1.52; *p* = 0.17), indicating no statistically significant difference in preoperative NLR between mortality and non-mortality groups ( Fig. 3 ). High heterogeneity was observed among the included studies ( *I* ^2^ = 89.45%). No significant outliers were identified based on studentized residuals and Cook's distance. There was no funnel plot asymmetry based on publication bias assessment (Egger's test *p* = 0.868; Begg's test *p* = 1.000). Leave-one-out sensitivity analyses were performed to evaluate the influence of individual studies on the pooled estimates. The overall direction of the effect remained consistent after exclusion of individual studies. However, omission of the study by Sharifi et al resulted in a statistically significant pooled effect, suggesting that this study contributed to the observed between-study heterogeneity. 22

**Fig. 4:**
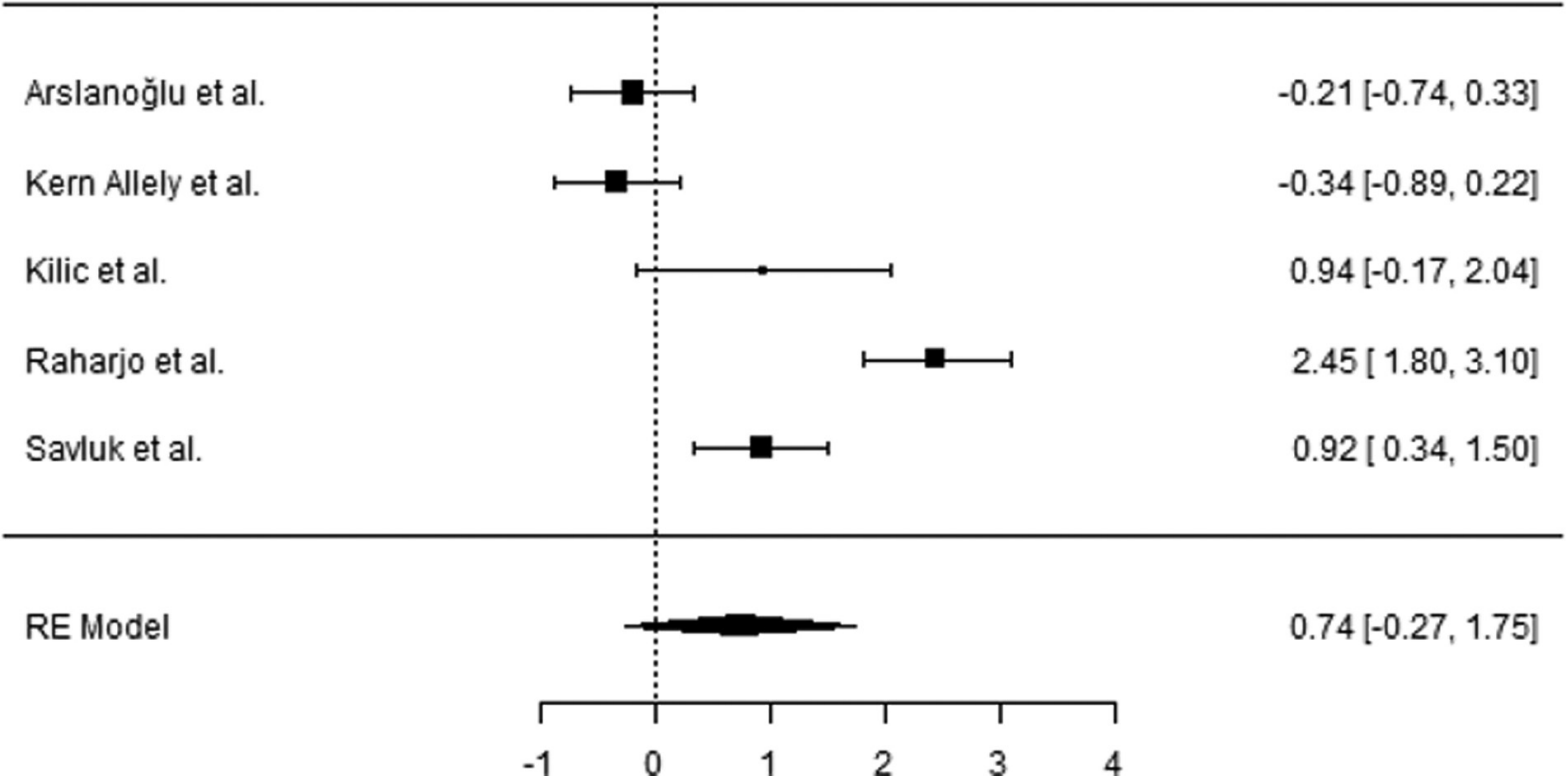
Forest plot of postoperative NLR and mortality. NLR, neutrophil-to-lymphocyte ratio.

For postoperative NLR, five studies were included in the postoperative analysis. The pooled SMD was 0.74 (95% CI: −0.27 to 1.75; *p* = 0.15), indicating no statistically significant difference between mortality and non-mortality groups. Heterogeneity was high ( *I* ^2^ = 91.99%). One study (Raharjo et al) was identified as a potential outlier; however, no study was deemed overly influential. 20 No significant publication bias was detected (Egger's test *p* = 0.619; Begg's test *p* = 0.483) ( Fig. 4 ). Leave-one-out sensitivity analyses were performed to evaluate the influence of individual studies on the pooled estimates. The sequential exclusion of each study did not alter the pooled effect size or its statistical significance.

### Correlation Between NLR and Postoperative Outcomes

Although NLR demonstrated a trend toward higher values in patients experiencing complications, the results were not statistically significant. Across all analyses, NLR did not show a statistically significant correlation with ICU length of stay, hospital length of stay, duration of mechanical ventilation, and postoperative complications.

Five studies evaluated the correlation between preoperative NLR and ICU length of stay. The pooled Fisher's *z* -transformed correlation coefficient was 0.39 (95% CI: −0.19 to 0.98; *p* = 0.19), indicating no significant association. Similarly, five studies assessed hospital length of stay. The pooled correlation coefficient was 0.43 (95% CI: −0.16 to 1.02; *p* = 0.15), showing no statistically significant relationship. Four studies assessed duration of mechanical ventilation. The pooled correlation coefficient was 0.43 (95% CI: −0.34 to 1.20; *p* = 0.28), indicating no significant association between preoperative NLR and duration of mechanical ventilation. Four studies were included in the analysis evaluating the overall postoperative complications. The pooled SMD was 0.598 (95% CI: −0.381 to 1.577; *p* = 0.231), indicating no statistically significant difference in postoperative NLR between patients with and without pulmonary complications. A total of six studies were included in the analysis evaluating the association between preoperative NLR and overall postoperative complications. The analysis showed a pooled SMD of 0.55 (95% CI: −0.09 to 1.18; *p* = 0.094), indicating no statistically significant difference between patients with and without postoperative complications ( Table 3 ).

**Table 3:** Summary of meta-analysis findings on perioperative NLR

Outcome	Effect size	95% CI	*p* -Value	Publication bias
Mortality (pre-op NLR)	SMD = 0.63	−0.27 to 1.52	0.17	Egger *p* = 0.868; Begg *p* = 1.000
Mortality (post-op NLR)	SMD = 0.74	−0.27 to 1.75	0.15	Egger *p* = 0.619; Begg *p* = 0.483
ICU length of stay (pre-op NLR)	*r* = 0.39	−0.19 to 0.98	0.19	Egger *p* = 0.623; Begg *p* = 0.817
Hospital length of stay (pre-op NLR)	*r* = 0.43	−0.16 to 1.02	0.15	Egger *p* = 0.318; Begg *p* = 0.817
Mechanical ventilation duration (pre-op NLR)	*r* = 0.43	−0.34 to 1.20	0.28	Egger *p* = 0.473; Begg *p* = 1.000
Postoperative complications (pre-op NLR)	SMD = 0.55	−0.09 to 1.18	0.09	Egger *p* = 0.117; Begg *p* = 1.000;

Abbreviations: CI, confidence interval; ICU, intensive care unit; NLR, neutrophil-to-lymphocyte ratio; SMD, standardized mean difference.

## Discussion

This systematic review assessed the clinical value of perioperative NLR in predicting mortality and adverse outcomes in pediatric patients undergoing CHD surgery and evaluated correlations between NLR levels and postoperative outcomes in this population. This systematic review found that NLR tends to be higher in patients with mortality and adverse outcomes compared to those without. However, the findings do not support a significant relationship between NLR and adverse outcomes in pediatric patients undergoing CHD surgery.

These higher levels of NLR indicate that patients in our included studies with worse outcomes may experience an increased inflammatory response compared to others. This suggests that the marker reflects the underlying inflammatory burden rather than serving as a definitive predictor of prognosis. The analysis showed no significant correlations between NLR and other postoperative outcomes, including ICU length of stay, hospital stay, duration of mechanical ventilation, and overall complications. The pathophysiology underlying these associations is likely multifactorial. Surgical trauma and CPB lead to a systemic inflammatory response, which leads to activation of neutrophils and relative lymphocyte suppression, resulting in elevated NLR levels. In pediatric patients, the immune system is immature and evolving, which leads to variability in inflammatory response and may explain the inconsistent associations observed in the studies included in our review. 28 29 30

In adult meta-analyses, perioperative NLR shows a statistically significant association with mortality and major adverse cardiac events, highlighting that elevated NLR reflects a clinically meaningful systemic inflammatory response in adults. 31 32 33 34 Meta-analyses in adults undergoing cardiac surgery have reported that an elevated perioperative NLR is independently associated with mortality, postoperative atrial fibrillation, and acute kidney injury, with pooled effect estimates reaching statistical significance despite high between-study heterogeneity. 31 32 33 34 The difference observed between adults and the pediatric population in our review may be explained by the more mature immune system in adults, where neutrophil activation and lymphocyte suppression follow a more predictable pattern in response to surgical stress, ischemia-reperfusion injury, and CPB. In pediatric patients, the immature immune system and variable inflammatory response may blunt the predictive value of NLR as observed in the studies included in the current analysis. 28 29 30 31 32 Adult studies are typically far larger and more homogeneous with respect to the underlying cardiac pathology (mostly ischemic heart diseases), which increases statistical power to detect modest effect sizes of the magnitude observed in our pooled estimates. Moreover, normal NLR values in children vary substantially with age, reflecting the physiological lymphocytosis of infancy and early childhood and its gradual shift toward an adult-like leukocyte profile later in childhood. Therefore, a fixed cut-off derived from an adult or mixed-age population is unlikely to perform consistently across the wide pediatric age range represented in our included studies.

In addition, postoperative and serial NLR measurements in our included studies were reported to have stronger associations with adverse outcomes compared to preoperative values. This suggests that NLR is reflecting acute inflammatory response following surgery rather than baseline patient condition. Similar observations have been reported in adult cardiac surgery, where postoperative inflammatory markers, including NLR, are associated with complications such as atrial fibrillation and mortality. 33 34

From a clinical perspective, NLR alone may not be sufficient as a standalone prognostic marker in pediatric cardiac surgery, as supported by the current findings. However, due to its low cost and wide availability, it can be used alongside other clinical and biochemical markers to improve risk stratification.

Our review has several limitations. A primary limitation is the substantial clinical heterogeneity across the included studies. The population encompassed a wide range of congenital heart defects of varying anatomical and physiological complexity, from anatomical septal defects to complex cyanotic lesions such as tetralogy of Fallot and hypoplastic left heart syndrome. Despite the variety of named defects, the included surgeries share a common physiological insult that includes surgical trauma, ischemia-reperfusion, and CPB. However, the substantial heterogeneity may limit the generalizability of the results, and the summary effect sizes should therefore be interpreted with caution. The moderate-to-high risk of bias noted in several studies is another limitation. Bias in the confounding/statistical-analysis domains likely reflects inadequate adjustment for key covariates and reliance on univariable analyses. Important variables such as surgical complexity, CPB duration, and comorbidities were not uniformly adjusted for, which may affect the results. In addition, some included studies had small sample sizes, which may reduce statistical power and contribute to nonsignificant findings. Limitations related to the review process include inclusion of only English-language studies, which may introduce language bias.

## Conclusion

This systematic review showed that perioperative NLR shows a trend toward higher values in patients with adverse outcomes but does not demonstrate a statistically significant association with mortality or complications. NLR reflects the inflammatory response but may not be sufficient as an independent predictor. Its utility may be greater as part of combined risk assessment strategies. Further high-quality studies are needed to clarify its role and improve its clinical applicability in pediatric cardiac surgery.
