# Supplementary material for: Prognostic Value of Neutrophil-to-Lymphocyte Ratio for Adverse Outcomes in Pediatric Congenital Heart Disease Surgery
Source: Avicenna J Med. 2026 Sep 9;16(3):116–24. doi: 10.1055/s-0046-1827862 (PMC13557809; doi:10.1055/s-0046-1827862)
Supplement: Supplementary file 1 [file 10-1055-s-0046-1827862-s260032.pdf]

Supplementary Material S1

Search Strategy

PubMed

Search string: ((“Neutrophil-to-Lymphocyte Ratio”[tw] OR “Neutrophil Lymphocyte Ratio”[tw] OR “Neutrophil to Lymphocyte Ratio”[tw] OR “NLR”[tw])) AND ((“congenital heart disease”[tw] OR “congenital heart defect”[tw] OR “CHD”[tw] OR “Cardiopulmonary Bypass”[tw] OR “Tetralogy of Fallot”[tw] OR “Hypoplastic Left Heart Syndrome”[tw] OR “Norwood Procedure”[tw] OR “Fontan”[tw] OR “Pediatric Cardiac Surgery”[tw]))

Web of Science

Search string: TS = ((“Neutrophil-to-Lymphocyte Ratio” OR “Neutrophil Lymphocyte Ratio” OR “Neutrophil to Lymphocyte Ratio” OR “NLR”) AND (“congenital heart disease” OR “Congenital Heart Defect” OR “CHD” OR “Cardiopulmonary Bypass” OR “Tetralogy of Fallot” OR “Hypoplastic Left Heart Syndrome” OR “Norwood Procedure” OR “Fontan” OR “Pediatric Cardiac Surgery”))

EMBASE

Search string: ((’neutrophil to lymphocyte ratio’:ti,ab OR ’neutrophil lymphocyte ratio’:ti,ab OR ’neutrophil-to-lymphocyte ratio’:ti,ab OR ’nlr’:ti,ab) AND (’congenital heart disease’:ti,ab OR ’congenital heart defect’:ti,ab OR ’chd’:ti,ab OR ’cardiopulmonary bypass’:ti,ab OR ’tetralogy of fallot’:ti,ab OR ’hypoplastic left heart syndrome’:ti,ab OR ’norwood procedure’:ti,ab OR ’fontan’:ti,ab OR ’pediatric cardiac surgery’:ti,ab))

ProQuest

Search string: ((ti,ab(“Neutrophil-to-Lymphocyte Ratio” OR “Neutrophil Lymphocyte Ratio” OR “Neutrophil to Lymphocyte Ratio” OR “NLR”))) AND ((ti,ab(“congenital heart disease” OR “congenital heart defect” OR “CHD” OR “Cardiopulmonary Bypass” OR “Tetralogy of Fallot” OR “Hypoplastic Left Heart Syndrome” OR “Norwood Procedure” OR “Fontan” OR “Pediatric Cardiac Surgery”)))

© 2026. The Author(s).
